# Prevalence and Temporal Distribution of Extrasystoles in Septic ICU Patients: The Feasibility of Predicting Fluid Responsiveness Using Extrasystoles

**DOI:** 10.1155/2018/5697092

**Published:** 2018-10-01

**Authors:** Johannes Enevoldsen, Cristhian Potes, Minnan Xu-Wilson, Simon T. Vistisen

**Affiliations:** ^1^Research Centre for Emergency Medicine, Institute of Clinical Medicine, Aarhus University, Aarhus, Denmark; ^2^Acute Care Solutions Department, Philips Research North America, Boston, MA, USA; ^3^Department of Anesthesia and Intensive Care, Aarhus University Hospitals, Aarhus, Denmark

## Abstract

**Background:**

Extrasystoles may be useful for predicting the response to fluid therapy in hemodynamically unstable patients but their prevalence is unknown. The aim of this study was to estimate the availability of extrasystoles in intensive care unit patients diagnosed with sepsis. The study aim was not to validate the fluid responsiveness prediction ability of extrasystoles.

**Methods:**

Twenty-four-hour ECG recordings from a convenience sample of 50 patients diagnosed with sepsis were extracted from the MIMIC-II waveform database, and ECGs were visually examined for correct QRS complex detection. Custom-made algorithms identified potential extrasystoles based on RR intervals. Two raters visually confirmed or rejected the potential extrasystoles and then classified them as ventricular, supraventricular, or unknown origin. Extrasystole availability was calculated as *extrasystolic coverage* for each 24 h ECG recording, that is, the percentage of the 24 h recording where an extrasystole had occurred in the preceding 30 minutes.

**Results:**

Mean *extrasystolic coverage* was 53.3% (confidence interval: [42.8; 63.6]%) and ventricular extrasystolic coverage was 21.4 [13.5; 29.8]%. Interrater reliability was strong for confirming/rejecting extrasystoles.

**Conclusions:**

Extrasystoles are available for fluid responsiveness prediction in septic patients in about half of the time. With this extrasystolic availability, we believe the method to be considered for clinical use, provided that future studies validate the method's fluid responsiveness prediction ability.

## 1. Introduction

Fluid administration is probably the most used treatment strategy when clinicians attempt to correct hemodynamic instability in critically ill patients. Fluids increase cardiac preload, and in turn increase cardiac stroke volume if the heart is operating on the steep part of the Frank-Starling curve. However, if the heart is operating on the plateau of the Frank-Starling curve the patient's heart will not respond with an increase in stroke volume when fluids are administered. Fluids have several side effects [1], which emphasize the need for methods that can predict fluid responsiveness, that is, whether or not fluid administration will improve hemodynamics.

Historically, several methods to predict fluid responsiveness have been suggested in the intensive care unit (ICU) setting. However, while intuitively useful, preload estimating variables such as central venous pressure are unreliable fluid responsiveness predictors [2]. The family of dynamic variables, for example, pulse pressure variation and stroke volume variation, constitutes reliable variables [2] but only in a narrow group of ICU patients who are deeply sedated and ventilated with specific ventilator settings [3]. When the tidal volume is low (below 7 ml/kg) or the patients have spontaneous breathing efforts, dynamic variables become unreliable [4–6]. Passive leg raising is a reliable alternative method [7], requires cardiac output monitoring, and is not a continuous measurement but requires an intervention, which also is the case for more recently suggested but less validated methods such as the end-expiratory occlusion test. For the vast majority of ICU patients, no variables from *continuous* monitoring offer broadly applicable and reliable fluid responsiveness prediction.

In the search for additional methods relying only on continuous monitoring, we have recently suggested that analysis of spontaneously occurring extrasystoles could be a novel method to predict fluid responsiveness [8]. The postectopic beat in the extrasystolic configuration is associated with an increased preload due to the compensatory pause and probably also due to a poor ejection at the ectopic beat. Analyzing the hemodynamic response at the postectopic beat, for example, how much systolic blood pressure changes compared with preceding sinus beats, may predict fluid responsiveness. Based on experimental data [9] as well as clinical data [8, 10], this method appears promising. While clinical validation in different patient groups is essential for considering the method's use in clinical practice, another crucial question arises for this specific method: to what extent are extrasystoles available in the everyday ICU setting? In postcardiac surgery patients, extrasystoles were available prior to a scheduled volume expansion in 61% of nonatrial fibrillation postcardiac surgery patients [8]. This figure may not be surprising when comparing with data for nonhospitalized older subjects, estimating the presence of extrasystoles in more than 97% of subjects (*n* = 1372) in a 24 h period and that, for example, more than 50% of elderly men (>80 years) have more than 15 hourly supraventricular extrasystoles. Still, extrasystolic occurrence was reported differently [11] and is difficult to interpret in this context. Fundamentally, it is unknown to what extent extrasystoles occur in an ICU population. If a patient has an extrasystole, it may be predictive of fluid responsiveness at that time, but the predictive value will surely decrease over time due to changes in the clinical setting, for example, treatment changes and illness deteriorations/improvements. Therefore, we have suggested that extrasystoles are useful in a 30-minute time window following the occurrence of the extrasystole [8].

The aim of this study was to estimate to what extent extrasystoles are available in septic patients in the ICU (based on the aforementioned 30-minute rule) and *not* to validate the method for its ability to predict fluid responsiveness. We investigated a convenience sample of septic patients, because sepsis patients constitute a patient category where fluid responsiveness prediction is of particular importance in the ICU [1].

## 2. Materials and Methods

### 2.1. Data Material

The analysis was based on continuous waveform recordings of ECGs from ICU patients who are *matched* to the MIMIC-II clinical database records, described in further detail at http://www.physionet.org [12]. The MIMIC database is a database approved by the US authorities waiving consent from the deidentified patients, whose data are in the database. The database is available for researchers worldwide. The selected dataset comprised 50 unique patients who were diagnosed with sepsis [13, 14] and had ECG (lead II) recorded for at least 24 hours continuously. The ECG recording duration was truncated to 24 hours to cover a full circadian cycle. The 50 waveforms were selected based on the consecutive order of the numeric subject IDs in the database. These subject IDs have historically been randomly assigned and are therefore not ordered with respect to the time of admission. We considered this approach most reproducible. Demographic and clinical characteristics for the entire remaining population in the matched clinical database (*n* = 2461) was also extracted for comparison including the subpopulation diagnosed with sepsis (*n* = 429). These extractions were not restricted to presence, type, or length of waveform monitoring. Only presence in the database (*n* = 2461) and sepsis diagnosis (*n* = 429) were extraction criteria. In case of several ICU admissions, the first ICU admission was selected for these patients. Vasopressor use was defined as administration of any of the following: norepinephrine, dopamine, epinephrine, vasopressin, isoprenaline, phenylephrine, or dobutamine.

### 2.2. Extrasystolic Coverage Definition

**Figure 1:**
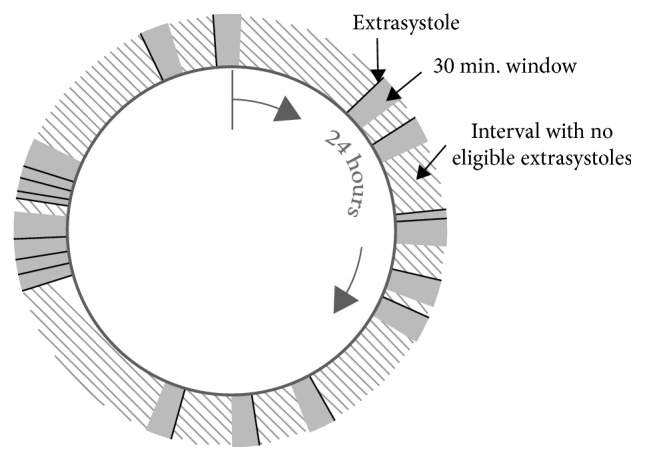
Illustration of the *extrasystolic coverage* definition. The fraction of time in which at least one extrasystole has occurred within the preceding 30 minutes. In this 24 h clock, the coverage graphically corresponds to the sum of the grey areas divided by the total 24 h area (grey areas plus shaded areas). In this illustration example, where 19 extrasystoles have occurred in 24 hours, extrasystolic coverage is approximately 30%, because the grey area corresponds to approximately 30% of the total area. The figure also illustrates how the 24 h recording is considered a “closed time loop,” that is, the last part of the recording is considered to precede the first part of the recording.

In the study, we define the term, *extrasystolic coverage*, as the fraction of time in a 24 h ECG recording, where at least one extrasystole has occurred within the preceding 30 minutes, see Figure 1 for further explanation.

### 2.3. Extraction of ECGs from MIMIC and R Spike Correction in Kubios HRV Software

Demographics and clinical characteristics were extracted from the MIMIC-II clinical database (records matched to the physiologic waveforms). Twenty-four-hour ECG data were converted from MIMIC's waveform database to a file readable to the publicly available software, Kubios HRV [15] (University of Eastern Finland, Kuopio, Finland). In cases where subjects had more than one 24 h record available from either a single admission with several monitoring days or multiple admissions, we included the chronologically first identified 24 h record.

The automatic R-wave detection in Kubios was visually inspected and corrected. For ventricular ectopic beats (where QRS morphology is altered), we marked the R-wave at the complex's largest deflection (i.e., whether positive or negative with respect to the isoelectric line). Initial visual inspection and correction in Kubios were done by one of two authors (JE or STV). ECGs with atrial fibrillation or other arrhythmia/issues precluding automatic detection for the entire 24 h period were termed *not analyzable* and assigned an extrasystolic coverage of 0%.

### 2.4. Detection of Potentially Eligible Extrasystoles

Corrected RR interval time series were automatically analyzed: RR intervals reduced by more than 20% compared with the preceding RR interval identified a potentially eligible extrasystole (the 20% limit ensures an altered preload at the subsequent postectopic beat [8]). Also, all ten RR intervals preceding a potentially eligible extrasystole had to be sinus beats (because they constitute a “baseline” for the method). We considered this criterion was met if none of the ten preceding RR intervals were more than 10% higher or lower than the median of the ten RR intervals (allowing presence of natural heart rate variability).

### 2.5. Manual Classification of Detected Potentially Eligible Extrasystoles

ECG segments surrounding potential extrasystoles (*n* = 10944) were visually classified by two individual raters (authors JE and STV). The inspection was organized by a custom script, presenting the ECG of each potential extrasystole (and surrounding heart beats) and awaiting the rater's classification into one of four categories: “Ventricular extrasystole,” “supraventricular extrasystole,” “extrasystole of unknown origin,” or “erroneous detection.” The criteria for classification as ventricular extrasystole were as follows:

1. Wide QRS complex (>120 ms)
2. Absence of P-wave
3. Altered T-wave morphology

In cases not meeting any of these criteria, the extrasystole was classified as supraventricular. In cases meeting some but not all criteria, it was a subjective decision to either specify the extrasystole as “ventricular” or “supraventricular” or classify as “extrasystole of unknown origin.”

The distinction between ventricular and supraventricular extrasystoles was done in case the origin of extrasystoles playing a role for the predictive power, even though the present clinical data at hand do not indicate this [8]. We report overall extrasystolic coverage and coverage for both subtypes of extrasystoles.

When calculating the *extrasystolic coverage*, extrasystoles occurring in the last 30 minutes of the recording were viewed as preceding the first 30 minutes of the ECG recording, that is, 24 hours was considered a “closed time loop” (also illustrated in Figure 1).

### 2.6. Statistics and Interrater Reliability Analyses

Clinical and demographic characteristics were compared with the chi-squared test for dichotomous variables or Wilcoxon's rank sum test for ordinal and continuous variables. Our cohort of 50 patients was compared with the remaining population in the clinical database diagnosed with sepsis (*n* = 429) as well as the entire remaining part of the matched database (*n* = 2461). In particular, available covariates that are known to affect extrasystolic occurrence [11, 16] were compared between the cohorts (age, gender, hypertension, and heart disease; heart disease was defined dichotomously as any presence of congestive heart failure, cardiac arrhythmia, valvular disease, pulmonary circulation disorders, or peripheral vascular disorders in the Elixhauser subitems, which are based on ICD9 and drug codes). Confidence intervals for extrasystolic coverage estimates were calculated using the percentile method on a bootstrap sample (10,000 resamples). We present extrasystolic coverage estimates for the entire cohort for the overall interpretation and comparison with, for example, dynamic variable applicability but also estimates for the analyzable subgroup (e.g., excluding atrial fibrillation records) to exclude cases where clinicians would not have expectations to the method.

Extrasystolic coverage's dependence on demographic and relevant clinical factors was analyzed in three multivariate linear regression models which all included gender, age, hypertension, and heart disease. One model additionally included *additional comorbidity* (Elixhauser sum score when omitting hypertension and heart disease items, as defined above) and *first SOFA score* (as measure of illness severity), another included time from ICU admission to start of ECG recording, and the third also included MAP, heart rate, and vasopressor use as available markers of hemodynamic state. Interrater reliability was analyzed for the four categories: “ventricular extrasystole,” “supraventricular extrasystole,” “extrasystole of unknown origin,” and “erroneous detection” using unweighted Cohen's kappa. Also, interrater reliability was calculated for the groups “any extrasystole type” and “erroneous detection.” A common categorization was subsequently made for disagreements. Initially consulting a cardiologist for ways to be more certain about extrasystolic type led to the conclusion that 12-lead ECG at a higher resolution would often have been necessary to determine the type in inconclusive cases. Extrasystolic coverage estimates are reported as mean [confidence interval (CI)]. Statistical testing was done in R 3.2.1 (R Core Team, 2015) or Matlab (version 2014a, MathWorks Inc., MA, USA).

## 3. Results

**Figure 2:**
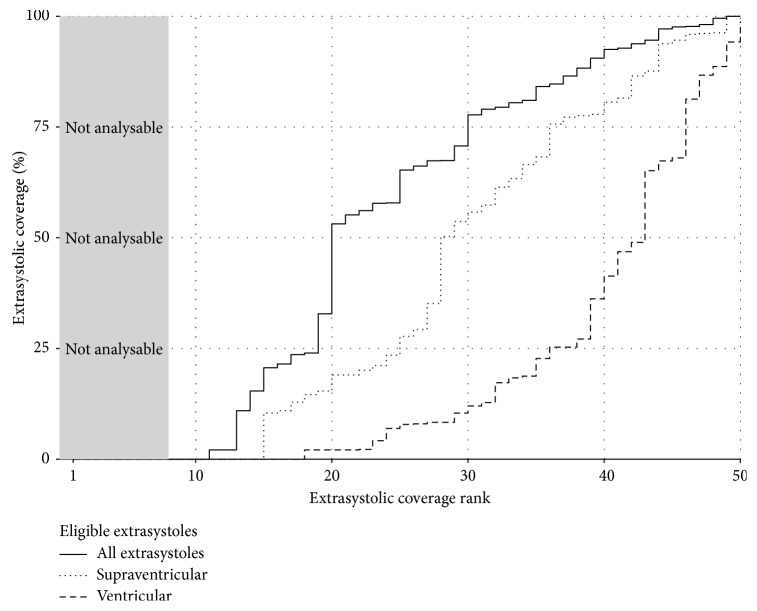
Visualization of extrasystolic coverage in the selected cohort. Solid line is the coverage of all extrasystoles (“supraventricular,” “ventricular,” and “of unknown origin”). Each “plateau” in the curves represents a patient's extrasystolic coverage. Each curve is sorted from lowest to highest extrasystolic coverage to make the areas under the curves easier to visualize and interpret; that is, the coverage numbers presented in the rightmost column of Figure 3 have been sorted (ranked) and plotted against the rank (1 up to 50). The area under each curve divided by the graph's entire area therefore corresponds to the estimated mean extrasystolic coverage across the patients (53.3%). If not-analyzable patients (the grey area) are excluded, the mean extrasystolic coverage is 63.5%.

**Figure 3:**
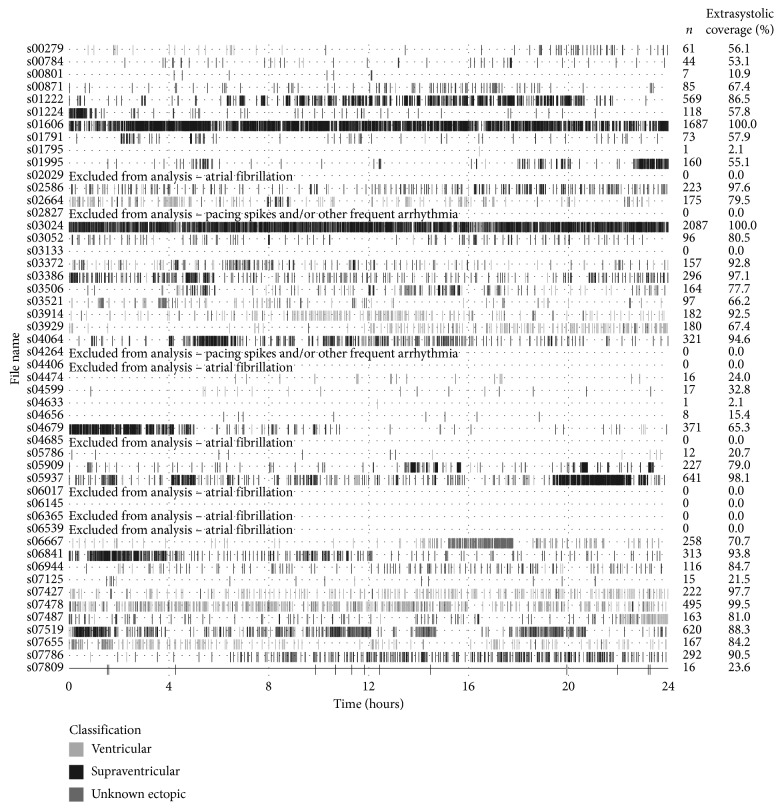
Extrasystolic coverage for each patient. Number (*n*) and extrasystolic coverage of eligible extrasystoles for each 24 h recording in the selected cohort (*n* = 50). The left column indicates the subject IDs in the MIMIC database.

Out of the 50 ECG recordings, 8 were not analyzable (6 due to atrial fibrillation and 2 due to pacing spikes and/or other frequent arrhythmia) resulting in forty-two 24 h ECGs for detailed ECG analysis. The *not-analyzable* records were assigned an extrasystolic coverage of 0%. Patient demographics and clinical characteristics are presented in Table 1. Generally, this cohort had characteristics not very different from the remaining septic patients in the matched subset. However, the selected 50 septic patients had significantly higher *first SOFA score* compared with the remaining septic patients. Across the 50-patient cohort, 53.3% [42.8; 63.6]% of the time was covered with extrasystoles according to the 30-min expiration period. Across the analyzable records, 63.5% [53.4; 73.1] of the time was covered with extrasystoles (illustrated in Figure 2). Looking at coverage for ventricular extrasystoles only, there was a coverage of 21.4 [13.5; 29.8]% in the 50-patient cohort and 25.4 [16.7; 34.9]% in the analyzable cohort 42 subjects. For supraventricular extrasystoles, the coverage was 41.1 [31.2; 51.5]% and 49.0 [38.2; 59.4]% respectively. Among the 42 analyzable records, all but two patients (95%) had at least one eligible extrasystole in the recording; 31 patients (73%) had at least 50% of the 24 h covered with extrasystoles; and 21 patients (50%) had at least 75% of the 24 h covered. In Figure 3, the number of eligible extrasystoles for each recording is shown along with the type and time of occurrence in the extracted 24 h recording. The median time from ICU admission to the beginning of the 24 h recording was 50.8, IQR [1.0; 144.6] hours (*n* = 48), with 21 recordings started during the first 24 hours after ICU admission. In multivariate linear regression analyses, age (*p* < 0.001) and heart disease (*p* < 0.05) were the only statistically significant factors influencing the extrasystolic coverage across all three models. *First SOFA score* (being the only difference between the selected cohort and the remaining septic patients) was not affecting extrasystolic coverage (*p*=0.82).

### 3.1. Interrater Reliability

Among 10,944 potential extrasystoles, 10,753 extrasystoles were visually confirmed and agreed upon by the two raters. In total, there were 919 disagreements (simple percentage agreement = 92%). Cohen's kappa for classification in four categories was 0.79. Most of these disagreements were systematic, that is, several extrasystoles with identical morphology for a specific patient where, for example, one rater repeatedly classified as *supraventricular* and the other classified as *unknown origin*. To assess the raters' ability to distinguish between eligible extrasystoles and erroneous detections, all types of extrasystoles (*supraventricular*, *ventricular*, and *ectopic of unknown origin*) were collapsed into a single category. This left 37 disagreements (simple percentage agreement = 99.7%). Kappa for classification in the two super categories (eligible extrasystole vs. erroneous detection) was 0.90.

## 4. Discussion

This is the first study to quantify the occurrence of extrasystoles in septic ICU patients, and extrasystoles were available for fluid responsiveness prediction in approximately half of the time. While not directly comparable, these figures stand in contrast to the dynamic variable usefulness of around 2–3% of ICU patients [3]. Excluding those patients where clinicians would not expect the extrasystoles method to be applicable (e.g., atrial fibrillation), 63.5% of the time was covered with extrasystoles. This is in accordance with a clinical validation study of the extrasystoles method in postcardiac surgery patients (61% had extrasystoles in the 30 min observation period) [8]. The indisputable primary limitation of dynamic variable use in the ICU is requirements to the preload varying mechanism: controlled mechanical ventilation mode, tidal volume, respiratory rate, compliance, etc. In theory, the extrasystoles method has no requirements to the way patients breathe. However, since any breathing pattern induces some (cyclic) variations in blood pressure, it may be important for the extrasystoles method to take into account *when* in a respiratory cycle, the postectopic beat occurs, which has the potential to improve the method compared with existing classification results [8, 10].

The interrater agreement on the classification of potential eligible extrasystoles was “moderate” to “strong” by common standards [17, 18]. In cases where the raters disagreed, the most conservative classification was predominantly chosen (i.e., “extrasystole of unknown origin” or “erroneous detection”). Of the 37 disagreements on whether or not a potential extrasystole was eligible, 26 (70%) were resolved by rating the potential extrasystole not eligible (“normal”). As such, a relevant proportion of these 37 disagreements appeared to be simple button press mistakes made by either of the raters, which is unrealistic to avoid when visually inspecting and manually classifying more than 10,000 potential extrasystoles. The 882 disagreements on the specific type of extrasystole were resolved by rating the potential extrasystole as “of unknown origin” in 841 (95%) of the cases. This has probably led to an underestimation of the frequency of the specific types of extrasystoles (i.e., ventricular and supraventricular shown in Figure 2).

We considered the criterion for preceding sinus beats met if none of the ten preceding RR intervals were more than 10% higher or lower than the median of the ten RR intervals preceding the potential ectopic beat. This was automatically verified by our customized software in advance of visual inspection. Since we after this confirmation visually verified the eligibility and origin of each extrasystole, we are certain that the high prevalence of extrasystoles for subjects such as s01606 in Figure 3 is indeed comprised by eligible extrasystoles and not associated with arrhythmia such as atrial fibrillation or coupled extrasystoles.

The primary limitation of the method is obviously that clinicians cannot wait for extrasystoles to occur. Nonetheless, the information held in the postectopic blood pressure configuration basically comes “free of charge” in patients monitored simultaneously with ECG and ABP but may be difficult to eyeball on today's monitors, considering that the optimal cut-off in, for example, systolic blood pressure change so far has been reported to be around 5% in a clinical setting [8]. The extrasystoles method is, theoretically, not restricted to a certain breathing pattern or lung compliance, but we *do* expect that the method has other limitations described for dynamic variables such as cardiac function and tamponade, so the coverage estimates presented here should be regarded as a maximal potential of the extrasystolic method. Still, if clinically validated, and based on this study's data, we believe that the extrasystolic method would be more applicable than dynamic variables in the ICU.

Regarding comparison of the clinical covariates that influence extrasystolic coverage, the selected cohort appeared reasonably representative of the remaining population diagnosed with sepsis in the matched clinical database (*n* = 429). The only striking difference, *first SOFA score* (a measure of illness/sepsis severity), did not affect extrasystolic coverage in our selected cohort (those analyzable, *n* = 42), and other clinical and demographic covariates that affect extrasystolic coverage (age, gender, hypertension, and cardiac disease) were not different between the selected cohort and the remaining septic patients. Still, the cohort was small, in particular for a multivariate regression and it could be reasonably argued that it is not powered for firm conclusions regarding the impact of first SOFA score on extrasystolic coverage. Yet, we believe that our estimates of extrasystolic coverage are qualified estimates given the otherwise good clinical resemblance between the selected cohort and the remaining population diagnosed with sepsis in the matched clinical database. We recognize sepsis severity could impact extrasystolic coverage, while it has not been described to influence the occurrence of extrasystoles. However, it has been shown that new-onset atrial fibrillation and thereby the ratio of nonanalyzable patients are related to the severity of sepsis, with patients in septic shock having a higher risk [19]. This was not found in our small sample, where only two of the 21 patients who received vasopressor treatment (9.5%) had atrial fibrillation against four of 29 who did not receive vasopressor treatment (13.8%). In addition to this, the extrasystolic occurrence estimate (53.3%) should be interpreted in the context of the relatively small sample size which is reflected in the estimated confidence interval [42.8; 63.6]%. In the planning of the study, we considered this cohort size adequate for rough estimation of the coverage and focused further on the validation of our classifications (interrater analyses). Inspecting more ECG records would lead to a narrower CI on the extrasystolic coverage estimate, but the current CI is indicative in our opinion and resembles what we have observed in clinical validation studies [8, 10].

As a final limitation to our study, sepsis criteria have been updated [20] during our data analyses and it is unknown if this would affect our extrasystolic coverage estimates.

## 5. Conclusion

The present study shows that extrasystoles are reasonably available for fluid responsiveness prediction in septic ICU patients. Overall, we believe that our systematic approach to detection, the final common classification along with strong agreement in the interrater reliability analysis, has resulted in a very reliable estimate of the real extrasystolic coverage in the selected cohort. It is up to clinicians to decide whether the glass is half full or half empty with the presented extrasystolic coverage (53% of the time). We think of it as half full because the information held in extrasystoles appears to predict fluid responsiveness and is readily available in half of ICU patients monitored with ECG and invasive arterial pressure. Still, a reasonably high prevalence/coverage of extrasystoles is not validating the prediction ability of this method, only rendering it feasible. Future validation studies on the extrasystoles method to predict fluid responsiveness in, for example, medical ICU patients and patients in general anesthesia have to be conducted before the extrasystolic method can be recommended for clinical use.

## Figures and Tables

**Table 1:** Demographics and clinical characteristics of the cohorts.

	Selected cohort (*n* = 50)	Matched subset with sepsis omitting selected cohort (*n* = 429)	Entire matched subset omitting selected cohort (*n* = 2461)
Age	68 (54; 76)	69 (52; 78)	68 (55; 79)
Male gender	25 (50%)	228 (53%)	1410 (57%)
*ICU admission type*			
(i) CCU	11 (22%)	146 (34%)	878 (36%)
(ii) CSRU	15 (30%)	126 (29%)	715 (29%)
(iii) MICU	24 (48%)	157 (37%)	854 (35%)
(iv) SICU	0 (0%)	0 (0%)	13 (1%)
(iv) NICU	0 (0%)	0 (0%)	1 (0%)
First SOFA score	10 (7; 13)	6 (3; 9)#	5 (2; 9)#
Elixhauser sum score	3 (2; 4)	2 (1; 4)	2 (1; 3)∗
*Comorbidity related to ES*			
(i) Heart disease	28 (56%)	200 (47%)	1019 (41%)∗
(ii) Hypertension	9 (18%)	108 (25%)	655 (27%)
*Primary diagnosis*			
Sepsis	15	NA	NA
Pulmonary	13	NA	NA
Cardiac function/arrhythmia	6	NA	NA
AMI	3	NA	NA
Hepatic	2	NA	NA
CNS	2	NA	NA
GI bleeding	1	NA	NA
Renal	3	NA	NA
Vasopressor use	21 (42%)	NA	NA
MAP (24 h mean)	75.7 (68.5; 84.8)	NA	NA
HR (24 h mean)	87.8 (74.9; 98.5)	NA	NA

Statistical comparisons are made between the selected cohort and the matched subset with sepsis (*n* = 429) and between the selected cohort and the remaining matched subset (*n* = 2461). Data are presented as median (interquartile range) for continuous and ordinal variables and as number (fraction of entire cohort in percent) for dichotomous variables. Primary diagnoses are reported with respect to the affected organ system, except for the sepsis diagnose. ^*∗*^*p* < 0.05 compared with selected cohort. ^#^*p* < 0.001 compared with selected cohort. CCU: Coronary Care Unit; CSRU: Cardiac Surgery Recovery Unit; MICU: Medical ICU; SICU: Surgical ICU; NICU: Neonatal ICU; GI: gastrointestinal; ES: extrasystoles; AMI: acute myocardial infarction; CNS: central nervous system; GI: gastrointestinal; MAP: Mean arterial pressure; HR: Heart rate; NA: not assigned.

## Data Availability

The study data are based on the publicly available database, MIMIC, accessible to researchers across the world.
